# Negative Pressure Wound Therapy in Closed Colorectal Surgical Incisions: A Systematic Review and Meta-Analysis

**DOI:** 10.7759/cureus.49621

**Published:** 2023-11-29

**Authors:** Nasser A AlJoaib, Faisal A Alghamdi, Batoul N AlEdwani, Abdulaziz K AlNaimi, Zeead M AlGhamdi

**Affiliations:** 1 Department of Medicine, Imam Abdulrahman Bin Faisal University, Dammam, SAU; 2 College of Medicine, King Saud Bin Abdulaziz University for Health Sciences, Jeddah, SAU; 3 Department of Thoracic Surgery, Imam Abdulrahman Bin Faisal University, Khobar, SAU

**Keywords:** negative pressure wound therapy, post-op wound complications, surgical site infections (ssi), closed incisional negative pressure wound therapy, lower gi or colorectal surgery

## Abstract

The utilization of negative pressure wound therapy (NPWT) in lowering the incidence of infections in closed colorectal surgical incisions has not been thoroughly established, and recent trials have had conflicting results. This meta-analysis was conducted to synthesize the findings of available trial data and carefully evaluate the effectiveness of this intervention in colorectal surgery. The databases PubMed, Embase, and Cochrane Central Register of Controlled Trials (CENTRAL) were combed for randomized controlled trials (RCTs) that compared negative pressure wound therapy to standard dressing in closed wounds of patients undergoing colorectal surgery. The incidence of surgical site infections (SSIs) was the primary outcome. Secondary outcomes included the occurrence of seroma and hematoma. The trial results were represented as odds ratios (OR) with a 95% confidence interval (CI), and a fixed-effects model was used. Nine studies found eligible were included, and the pooled results revealed that negative pressure wound therapy significantly reduced the incidence of surgical site infections (OR: 0.70; 95% CI: 0.53, 0.93; P= 0.02). Furthermore, there was a significant reduction in seroma (OR: 0.27; 95% CI: 0.08, 0.95; P = 0.04) and hematoma (OR: 0.20; 95% CI: 0.04, 0.96; P = 0.04). The use of negative pressure wound therapy for primarily closed incisions has been increasing, and our results indicate that it is superior to standard surgical dressings in preventing surgical site infections and other wound complications in colorectal surgeries.

## Introduction and background

The Centers for Disease Control and Prevention (CDC) coined surgical site infection (SSI) in 1992, and its definition encompasses incisional SSI and organ/space SSI [1]. SSIs are a significant and common complication of surgery, accounting for 20% of health-associated infections and occurring in 2% of all surgical procedures [2]. They commonly lead to a decreased quality of life (QoL) for the patient, a prolonged treatment course, an increased hospital length of stay (LOS), and high healthcare expenses [3]. Due to the nature of colorectal procedures and the considerable bacterial load associated with them, they are considered to have a high risk of SSI, with reports reaching as high as 26% [4].

Numerous strategies have been implemented to prevent SSIs, including antibiotic-impregnated dressings and incisional closure techniques [4]. Negative pressure wound therapy (NPWT), a therapeutic approach involving the application of subatmospheric pressure to wounds, has emerged as a promising tool for minimizing surgical site infections (SSIs) and surgical wound complications (SWCs) in primarily closed surgical incisions. The main applications of NPWT have traditionally been on open wounds to help accelerate healing in wounds treated by secondary intention; however, for wounds treated by primary intention, NPWT is increasingly being explored and is hypothesized to increase tissue perfusion and tensile strength and minimize drainage [5].

A 2020 systematic review with 5693 patients examined the use of NPWT in wounds from several surgical procedures and reported that NPWT reduced SSIs considerably compared to standard care [6]. In the context of colorectal surgery, however, the outcomes are inconsistent. A recent randomized controlled trial (RCT) on NPWT for colorectal surgery demonstrated no difference between NPWT and standard dressings [7]. Another RCT that evaluated NPWT in stoma reversal surgery found that it significantly reduced SSIs and SWCs [8].

No systematic reviews on the subject of colorectal surgery have been conducted. This meta-analysis seeks to systematically synthesize and evaluate existing research on the use of NPWT for preventing SSIs and other wound complications in primarily closed wounds in patients undergoing colorectal surgery.

## Review

Methods

This analysis was conducted as per the Preferred Reporting Items for Systematic Reviews and Meta-Analyses (PRISMA) [9]. This review was registered with the International Prospective Register of Systematic Reviews (PROSPERO) as number CRD42023408087.

Data Sources and Search Strategy

A search was conducted of the electronic databases PubMed, Embase, and Cochrane Central Register of Controlled Trials (CENTRAL) from their inception until March 2023. There were no language restrictions. To identify additional studies, the reference lists of relevant articles and conference proceedings were manually searched. The search strategy for PubMed is as follows: ("Negative pressure wound therapy" OR "negative pressure dressing" OR "negative pressure dressing [MeSH Terms]" OR "closure, vacuum assisted [MeSH Terms]" OR "vacuum assisted closure" OR "VAC" OR "NPWT") AND ("outcome" OR "outcomes" OR "SSI" OR "surgical site infection*" OR "surgical wound complication*" OR "infection, surgical wound [MeSH Terms]").

Study Selection

Studies were included if they were randomized controlled trials comparing NPWT to standard dressings in colorectal surgery patients. Another inclusion criterion was whether surgical site infections were reported as primary or secondary outcomes. The exclusion criteria were if the studies involved non-colorectal surgical procedures, included the use of NPWT for open incisions, and did not report surgical site infection as an outcome.

The literature search results were exported to the EndNote reference library software (Clarivate Plc, London, United Kingdom). After screening for and removing duplicates, the articles that remained were assessed by two independent reviewers (NA and FA), and the trials meeting the inclusion criteria were chosen. Based on the titles and abstracts, the studies were further evaluated, followed by a comprehensive full-article review to confirm their applicability. To resolve any discrepancies, a third reviewer (BA) was consulted.

Data Extraction and Study Quality Assessment

Two reviewers (ZA and AA) independently extracted study and patient characteristics, and a third reviewer was consulted for any discrepancies (NA). The following data was extracted: study characteristics (author, publication year, and study design), participant characteristics (sample size, age, and sex), and the type of surgery and NPWT device used. The following outcomes were extracted: the incidence of SSI, hematoma, and seroma. The Cochrane Collaboration's Risk of Bias tool was used to evaluate the risk of bias in the included trials.

Statistical Analysis

For the statistical analysis, the Cochrane Collaboration's Review Manager (version 5.4.1; Copenhagen, Denmark) was utilized. The outcomes of interest were combined using a fixed-effects model, and the results were reported as odds ratios (OR) with 95% confidence intervals (CIs) for dichotomous outcomes and as mean differences (MD) with 95% CIs for continuous outcomes. To evaluate heterogeneity, the Higgins I^2^ statistic was utilized, and a value of less than 50% was regarded as acceptable. In every instance, a P value of 0.05 or less was considered statistically significant.

Results

Literature Search Results

**Figure 1:**
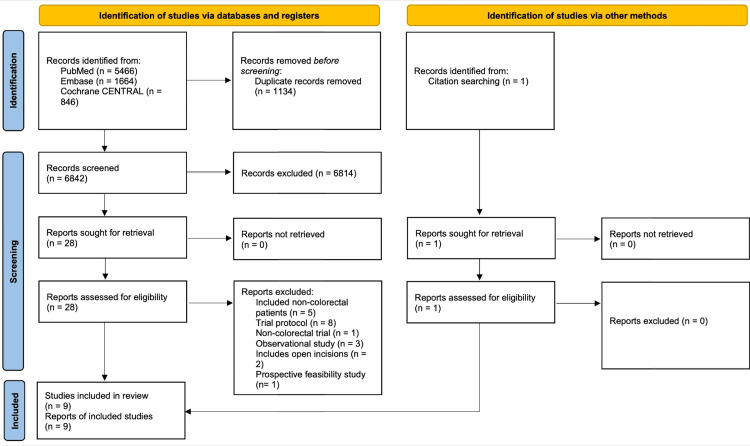
PRISMA flow diagram for the literature search across three databases and other sources. PRISMA, Preferred Reporting Items for Systematic Reviews and Meta-Analyses; CENTRAL, Cochrane Central Register of Controlled Trials

The search of the three electronic databases yielded a total of 7976 relevant studies. The PRISMA flow chart, as depicted in Figure 1, provides an overview of the results of our comprehensive literature search. Following the deletion of duplicate studies, the titles and abstracts of a total of 6842 studies were evaluated. In the end, 28 articles were retrieved for full-text review, of which eight met the inclusion criteria and were included in the analysis, and one study was obtained from citation searching.

Study Characteristics

The included trials randomly assigned a total of 1273 patients undergoing colorectal surgery, with 647 in the NPWT group and 626 in the control group. Table 1 presents the study characteristics of all included trials, including participant demographics and intervention details.

**Table 1:** Study characteristics of the included trials: the presentation of numerical data is either as a number (n) or as a mean and standard deviation (SD). RCT, randomized controlled trial; NPWT, negative pressure wound therapy; SSI, surgical site infection; ECF, enterocutaneous fistula; NA, not available; VICNISS, Victorian Nosocomial Infection Surveillance System

Study name, year	Study design	Negative pressure therapy used	Type of surgery (NPWT group), n	Type of surgery (control group), n	Population, n	Sex, n (male/female)	Age, mean (SD)	Definition of SSI	Outcomes reported
Arellano et al., 2021 [10]	RCT	Prevena Incision Management System (Kinetic Concepts, San Antonio, TX)	Elective, 69; emergency, 6	Elective, 57; emergency, 16	NPWT, 75; control, 73	85/63	NPWT, 71.7 (10.877); control, 69.1 (11.73)	Centers for Disease Control and Prevention criteria	Incidence of SSI
Carrano et al., 2021 [11]	RCT	PICO™ (Smith and Nephew, Hull, United Kingdom)	Ileostomy closure, 38; colostomy closure, 7; ileorectal anastomosis, 3; Hartmann's reversal, 2	Ileostomy closure, 27; colostomy closure, 6; ileorectal anastomosis, 11; Hartmann's reversal, 4	NPWT, 49; control, 45	66/32	NPWT, 56.32 (12.92); control, 55.08 (16.25)	Centers for Disease Control and Prevention criteria	Incidence of SSI and hematoma
Flynn et al., 2020 [12]	RCT	PICO™ (Smith and Nephew, St. Petersburg, FL)	(Laparotomy) Small bowel, 5; right colon, 25; left colon, 11; rectum, 41; colostomy, 14	(Laparotomy) Small bowel, 2; right colon, 19; left colon, 20; rectum, 39; colostomy, 12	NPWT, 96; control, 92	111/77	NPWT, 64.2 (13.2); control, 66.8 (13.3)	VICNISS definition based on the Centers for Disease Control and Prevention model	Incidence of SSI, hematoma, and seroma
Kaçmaz et al., 2022 [8]	RCT	PICO™ (Smith and Nephew, Fort Worth, TX)	Stoma closure, 4; right hemicolectomy, 4; left hemicolectomy, 2; low anterior resection, 12	Stoma closure, 8; right hemicolectomy, 4; left hemicolectomy, 3; low anterior resection, 8	NPWT, 24; control, 26	27/23	NPWT, 67.4 (9.1); control, 64.5 (9.1)	Centers for Disease Control and Prevention criteria	Incidence of SSI and seroma
Leon et al., 2016 [13]	RCT	NA	Open colorectal surgery: 47	Open colorectal surgery: 34	NPWT, 47; control, 34	NA	NA	NA	Incidence of SSI
Murphy et al., 2019 [14]	RCT	Prevena Incision Management System (Kinetic Concepts, San Antonio, TX)	Right colon resections, 37; left colon resections, 36; subtotal colectomy, 20; low anterior/rectal, 39; converted to open, 26; ostomy, 48	Right colon resections, 38; left colon resections, 47; subtotal colectomy, 12; low anterior/rectal, 43; converted to open, 17; ostomy, 51	NPWT, 144; control, 140	154/130	NPWT, 64 (15); control, 64 (15)	Centers for Disease Control and Prevention criteria	Incidence of SSI
Sapci et al., 2023 [7]	RCT	Prevena Incision Management System (Kinetic Concepts, San Antonio, TX)	Colectomy, 43; ECF takedown, 16; pouch revision, 35; proctectomy, 18; proctectomy and pouch, 18; small bowel resection, 19	Colectomy, 50; ECF takedown, 2; pouch revision, 32; proctectomy, 15; proctectomy and pouch, 14; small bowel resection, 17	NPWT, 149; control, 149	151/147	NPWT, 49.8 (16.4); control, 52.2 (15.1)	Centers for Disease Control and Prevention criteria	Incidence of SSI
Uchino et al., 2016 [15]	RCT	PICO™ (Smith and Nephew, Hull, United Kingdom)	Elective ostomy closure: 31	Elective ostomy closure: 28	NPWT, 28; control, 31	40/19	NPWT, 48.1 (14.9); control, 40.4 (15.9)	Centers for Disease Control and Prevention criteria	Incidence of SSI
Wierdak et al., 2021 [16]	RCT	Nanova™ (3M Medical, Maplewood, MN)	Elective ileostomy closure: 35	Elective ileostomy closure: 36	NPWT, 35; control, 36	44/27	NPWT, 61.6 (11.3); control, 62.4 (11.3)	Centers for Disease Control and Prevention and European Centre for Disease Prevention and Control criteria	Incidence of SSI, hematoma, and seroma

Quality Assessment and Publication Bias

**Figure 2:**
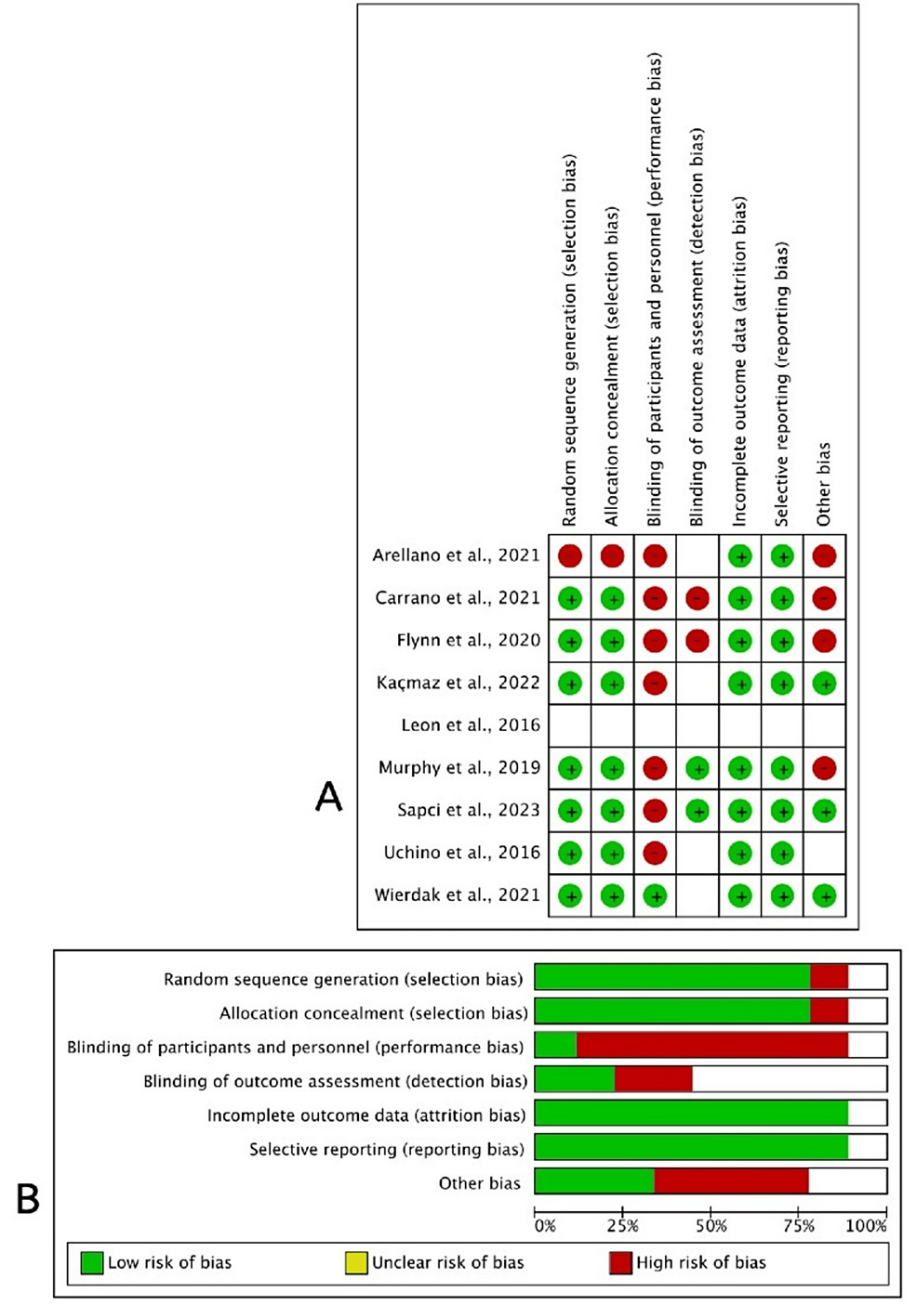
(A) Risk of bias summary: the opinions of the review authors regarding each risk of bias item for every trial that was included. (B) Risk of bias graph: review authors' evaluations of each risk of bias item depicted as percentages throughout all included trials.

The quality assessment of the included studies is provided in detail in Figure 2A, 2B. The majority of the included studies had high methodological quality in terms of random sequence generation, allocation concealment, outcome data, and reporting. However, a high risk of bias was observed in the blinding domains owing to the nature of the intervention. A high level of bias was observed in the other bias domain, as three studies were funded by the manufacturers of the NPWT devices.

Results of Meta-Analysis

**Figure 3:**
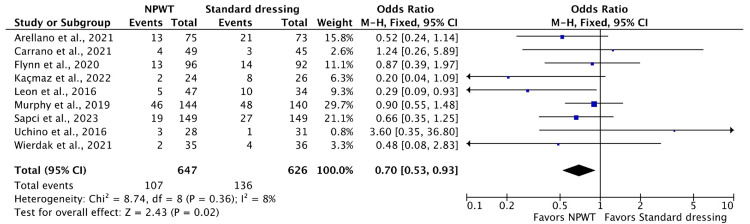
Forest plot demonstrating the effect of negative pressure wound therapy (NPWT) as compared to standard dressings in preventing the incidence of surgical site infections (SSI). CI, confidence interval; df, degrees of freedom; M-H, Mantel-Haenszel

**Figure 4:**
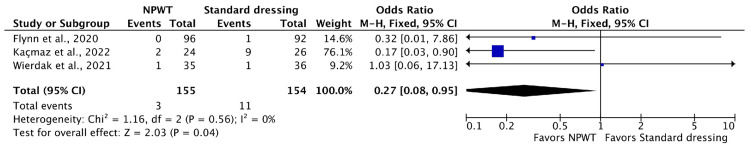
Forest plot showing the effect of negative pressure wound therapy (NPWT) compared to standard dressing in reducing the incidence of seroma. CI, confidence interval; df, degrees of freedom; M-H, Mantel-Haenszel

**Figure 5:**
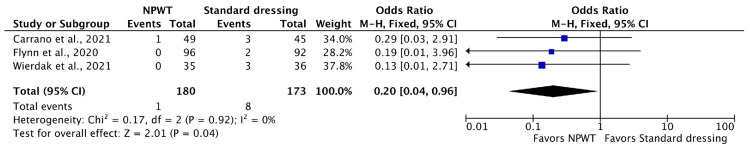
Forest plot demonstrating the effect of negative pressure wound therapy (NPWT) as compared to standard dressings for preventing the incidence of hematoma. CI, confidence interval; df, degrees of freedom; M-H, Mantel-Haenszel

The results of our meta-analysis are presented in Figures 3-5. Forest plots with the effect size of each in detail are given.

SSI at 30 days: Out of the nine included studies (Figure 3), all of them reported the incidence of SSI (NPWT, 647 patients and 107 events; control, 626 patients and 136 events). NPWT significantly reduced the incidence of SSI following colorectal surgery at 30 days when compared to standard surgical dressing (OR: 0.70l, 95% CI: 0.53, 0.93; P = 0.02).

Incidence of seroma: Three studies (Figure 4) reported the incidence of seroma (NPWT, 155 patients and three events; control, 154 patients and 11 events). The use of NPWT significantly reduced the occurrence of seroma in colorectal surgery patients in contrast to conventional dressing (OR: 0.27; 95% CI: 0.08, 0.95; P = 0.04).

Incidence of hematoma: Three studies (Figure 5) reported data on the occurrence of hematoma (NPWT, 180 patients and one event; control, 173 patients and eight events). NPWT was favored over standard dressing as it significantly reduced the incidence of hematoma in patients following colorectal surgeries (OR: 0.20; 95% CI: 0.04, 0.96; P = 0.04).

Discussion

Throughout history, the importance of wound care has been emphasized since the early days of surgery, encompassing various aspects such as surgical treatment, debridement, and hemostasis [17]. Recent advancements in wound closure techniques have focused on efficiently achieving wound healing with minimal complications. These innovations aim to improve the overall outcome of the healing process [18]. This study aimed to compare negative wound pressure therapy and its effectiveness to standard dressing in patients undergoing colorectal surgery with closed surgical incisions. We included nine trials with a total of 1273 patients, of which 647 were in the NPWT group and 626 were in the control group.

The global incidence of SSI after appendectomy is a significant concern, particularly in low-income countries where resources for infection control are limited, leading to adverse patient outcomes. A meta-analysis revealed an overall SSI incidence of seven per 100 patients, with the highest rates observed in Africa [19]. Recognizing the gravity of SSIs, the World Health Organization (WHO) has outlined 16 recommendations for prevention, including the use of NPWT in closed surgical incisions, albeit with low-quality evidence [20].

While our analysis suggests a potential reduction in SSI incidence with NPWT compared to standard surgical dressings, it is crucial to acknowledge the complexity introduced by heterogeneity in surgical indications, procedural variations, and differences between elective and acute procedures across selected studies. These inherent limitations should be considered when interpreting our findings. Notably, our observations align with another meta-analysis involving 6624 patients, reporting a decreased risk of SSI associated with NPWT compared to the control group [21]. However, caution is warranted in drawing firm conclusions due to the varied nature of the included studies.

Inconsistencies in the literature regarding the effectiveness of NPWT for closed laparotomy incisions have been noted. Another meta-analysis on this topic found no significant difference between NPWT and standard care, although the pooled estimate approached significance [22]. Moreover, a separate meta-analysis with a significantly larger and broader sample size of 3193 patients concluded that a definitive recommendation on the prophylactic usage of NPWT for the prevention of SSI cannot be made. While a statistically significant association was found between a decrease in site infections and NPWT prophylactic usage, the findings were limited by high heterogeneity, potentially attributed to the inclusion of nonrandomized observational studies [23]. These conflicting results and the presence of high heterogeneity underscore the complexities of drawing definitive conclusions. We recognize the need for further research and emphasize caution in interpreting the current state of evidence.

Similarly, the incidence of seromas and hematomas was also significantly reduced in patients who had undergone NPWT in comparison to standard dressing. These findings correlate with a meta-analysis involving 1858 patients with various closed surgical wounds [24]. On the other hand, a previously mentioned meta-analysis conducted a comparative analysis of the rates of seroma in both the NPWT group and the standard dressing group and found no significant difference. However, in their analysis of seroma, three of the four studies were observational studies, which are considered inferior in terms of hierarchical research evidence compared to RCTs [23].

It is believed that NPWT reduces complications primarily through alterations in perfusion caused by an increase in angiogenesis [25]. Increased blood flow improves tissue delivery of oxygen and nutrients, as well as debris removal. Another essential mechanism is the formation of granulation tissue through the application of a wound filler such as foam or gauze; the constant pressure allows the cells to enter the filler's pores, thereby increasing proliferation [26]. It is hypothesized that exudate control, in which excessive interstitial fluid is removed, promotes healing with local changes in blood flow and the elimination of toxic compounds [27].

In our comprehensive analysis, the studies included in our meta-analysis share common limitations that necessitate careful consideration. Challenges encompass potential type II errors, the absence of cost analysis, and difficulties in studying high-risk populations. Additionally, limitations related to small sample sizes affecting statistical power and challenges in delineating specific patient groups benefiting from the intervention were observed across studies. Multisite studies encountered issues with standardization, differences in usual care, and variations in patient enrollment. Further constraints included sample size limitations, open-label designs, and the imperative for future research with larger populations or high-risk patients. Other limitations, such as patient exclusions, crossover between groups, and a lack of detailed classification of wound infections, were also acknowledged.

Furthermore, the predominantly developed country setting of these studies, characterized by abundant resources and stringent infection control policies, raises considerations about the generalizability of the results. Moreover, a notable subset of trials, approximately a third, introduce substantial bias risks due to funding from NPWT manufacturers. This underscores the need for cautious interpretation and emphasizes the call for further research. Larger prospective studies with extended follow-up are warranted to fully evaluate the effectiveness of NPWT in colorectal surgery, considering these collective limitations.

## Conclusions

Our study results demonstrate a reduction in SSI. However, it is crucial to acknowledge that firm conclusions are challenging due to the heterogeneity between studies and inherent limitations. The variations in study designs and potential biases within the included studies necessitate a cautious interpretation of our findings. Further research, addressing these limitations and exploring the sources of heterogeneity, is imperative for a more robust understanding of the impact of NPWT on SSI reduction.
